# Genotoxic Effect in Autoimmune Diseases Evaluated by the Micronucleus Test Assay: Our Experience and Literature Review

**DOI:** 10.1155/2015/194031

**Published:** 2015-08-03

**Authors:** Olivia Torres-Bugarín, Nicole Macriz Romero, María Luisa Ramos Ibarra, Aurelio Flores-García, Penélope Valdez Aburto, María Guadalupe Zavala-Cerna

**Affiliations:** ^1^Facultad de Medicina, Universidad Autónoma de Guadalajara, 45129 Zapopan, JAL, Mexico; ^2^CUCBA, Universidad de Guadalajara, 45221 Guadalajara, JAL, Mexico; ^3^Unidad Académica de Medicina, Universidad Autónoma de Nayarit, 63155 Tepic, NAY, Mexico

## Abstract

Autoimmune diseases (AD) are classified into organ-specific, systemic, and mixed; all forms of AD share a high risk for cancer development. In AD a destructive immune response induced by autoreactive lymphocytes is started and continues with the production of autoantibodies against different targets; furthermore apoptosis failure and loss of balance in oxidative stress as a consequence of local or systemic inflammation are common features seen in AD as well. Micronucleus (MN) assay can be performed in order to evaluate loss of genetic material in a clear, accurate, fast, simple, and minimally invasive test. The MN formation in the cytoplasm of cells that have undergone proliferation is a consequence of DNA fragmentation during mitosis and the appearance of small additional nuclei during interphase. The MN test, widely accepted for *in vitro* and *in vivo* genotoxicity research, provides a sensitive marker of genomic damage associated to diverse conditions. In here, we present a review of our work and other published papers concerning genotoxic effect in AD, identified by means of the MN assay, with the aim of proposing this tool as a possible early biomarker for genotoxic damage, which is a consequence of disease progression. Additionally this biomarker could be used for follow-up, to asses genome damage associated to therapies.

## 1. Introduction

The immune system functions in order to maintain an organism survival against a threat through recognition of pathogen derived antigens and the conduction of an immune response to eliminate them; for that purpose the innate and adaptive immunity conduct organized and effective responses. Nevertheless, these mechanisms are not always accurate, sometimes a poor response may develop as seen in chronic infections, the recognition of self-proteins initiates autoimmunity, and the presence of an exaggerated Th2 type of response is associated with allergic conditions [1]. From a clinical point of view, autoimmune diseases (AD) are characterized by the presence of autoantibodies that can lead to tissue damage and destruction. Mechanisms in autoantibody generation can be explained through a loss of tolerance, but how does tolerance brake up occurs? In most instances mechanisms that initiate this loss of tolerance are unknown, but several studies suggest the participation of environmental, genetic and infectious factors. AD can be classified as organ-specific or systemic, depending on whether the autoimmune response is directed against a particular tissue as has been described in chronic lymphocytic thyroiditis (Hashimoto's thyroiditis) where circulating antibodies against thyroid peroxidase and thyroglobulin can be found [2], or against widespread antigens such as anti-nuclear antibodies in systemic lupus erythematosus (SLE) [3].

Autoantibodies production then has a key role in AD development and is also used as a disease marker for classification, evaluation, and monitoring disease activity. One of the most studied is antinuclear antibodies (ANA) detected mainly in patients with SLE but also found in patients with other AD. Antinuclear antibodies are immunoglobulins that react against different nuclear (ssDNA, dsDNA, SSA/Ro, etc.) and cytoplasmic components (aminoacil tRNA synthetase, Jo-1, mitochondria, etc.). Autoantibodies and other clastogenic factors (CF) have been identified in AD, capable of inducing chromosomal breakage [4]. For example, the formation and scavenging activity of free radicals in biological systems have been closely linked to a number of pathological conditions, and there is increasing evidence that ROS and the resulting prooxidant/antioxidant imbalance play a major role in AD [5, 6].

**Figure 1:**
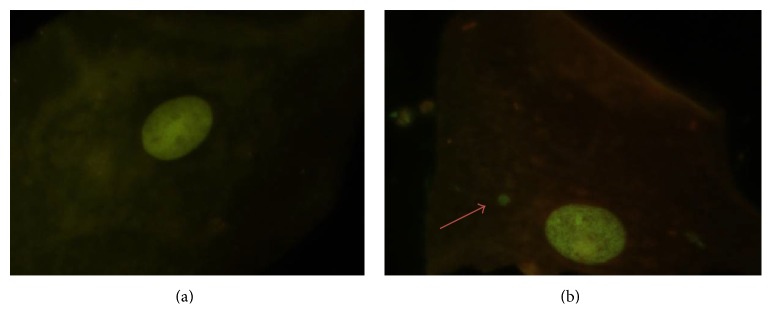
(a) Buccal mucosa cell with normal nuclei and (b) micronucleated cell from buccal mucosa. Photomicrographs stained with acridine orange viewed at 1000 magnification under fluorescence with an IVFL filter (450–490 nm). Binocular Microscope Carl Zeiss (Axiostar Plus). Sample was collected after a gentle swab of both cheeks oral mucosa with a polished slide; then the samples were spread directly in two separated slides previously identified, fixed with 80% ethanol, and stained for analysis; the sample must contain between 500 and 4000 cells to proceed with MN quantification [16, 17].

Genomic damage is probably the most important fundamental cause of degenerative disease. It is also well established that genomic damage can be produced by environmental exposure to genotoxic factors, medical procedures (e.g., radiation and chemicals), micronutrient deficiency (e.g., folate), lifestyle (e.g., alcohol, smoking, drugs, and stress), and genetic defects, such as inherited defects in DNA metabolism and DNA repair [7]. It is essential to have a reliable and minimally invasive biomarker to improve monitoring, diagnostics, and treatment in AD. The micronucleus (MN) assay is an excellent biomarker candidate, since it is able to detect chromosome breakage or malfunction of mitotic spindle caused by aneugenic mechanisms [8]. MN are formed during mitosis when lagging chromosomes are left out the metaphase plate due to mitotic defects (aneuploid effect) or when chromosome fragments lacking a centromere are not captured by spindle microtubules (clastogenic effect); in both cases the genetic material was unable to be incorporated into the nucleus of daughter cells [9]. The aneuploid effect can be distinguished from the clastogenic damage by differences in MN size [10] or by the presence of a centromere [11]. Both events can develop spontaneously; nevertheless their frequency can be increased under exposition to certain endogenous or exogenous agents [4, 12, 13]. Quantification of MN frequency is easily performed in any tissue that is undergoing constant proliferation [14], like oral mucosa epithelia [7, 15, 16] (Figure 1), or in lymphocytes by two different assays [17]. With respect to oral mucosa, it has been described that MN are observed in the basal layer of epithelial tissue and their presence can reflect genotoxic damage occurred up to 14 days prior to sample collection [7, 15]. For all of these reasons, MN frequency can be considered as a biomarker for mutagenic and genotoxic damage [14]. To further support this notion we aimed to perform a review of the literature that included information about MN frequencies in different AD.

## 2. Methods

We reviewed data from the available medical literature. A systematic search of papers was performed from data bases PUBMED, Clinical Key Elsevier, and ProQuest. We used the following search terms: “Autoimmune diseases” and “Micronucleus.” We included all papers in English and Spanish, obtained with dates from 1995 until 2014.

## 3. Results and Discussion

We identified 25 research papers: 17 were performed in lymphocytes, 7 were performed in buccal mucosa, and 1 was performed in fibroblasts. MN frequency was associated with different pathological conditions listed in Table 1.

### 3.1. Organ Specific Autoimmune Disease

We found 3 publications about Graves' disease, 2 studies related to type 1 diabetes mellitus (T1DM), 2 studies in multiple sclerosis, and 2 with affections of the skin, one in vitiligo, and one more in psoriasis patients.

Graves' hyperthyroidism is an autoimmune thyroid disease in which autoimmunity against thyroid antigens develops. The major antigen in Graves' disease (GD) is the thyroid-stimulating hormone receptor (TSHR), but antibodies against thyroid peroxidase (TPO) and thyroglobulin (Tg) can also be identified in up to 70% of patients with GD [36]. Radioiodine therapy is the treatment of choice for recurrent hyperthyroidism; in radiation exposed patients, chromosomal damage may occur due to an increase in superoxide radicals (clastogenic factors). After literature search, we did not identified publications that compare MN frequencies between untreated GD patients and healthy controls; it would have been desirable to state such differences for further analyzing the effect of specific treatments. The study publications that we found are related to the evaluation of micronuclei formation after ^131^I treatment in GD patients [18, 19] and hyperthyroid patients (GD, toxic multinodular goiter and autonomous toxic nodule) [20]. In the study performed by Ballardin et al. [18], it was found that, in the hours immediately following the administration of radioiodine, a modification in the oxidative equilibrium occurs, as documented by reduction in vitamin E levels, followed by the appearance of genetic damage, shown by a twofold to threefold increase in MN frequency in observed lymphocytes. Authors concluded that MN formation in peripheral lymphocytes after radioiodine therapy correlated with the release of clastogenic factors in the plasma and negatively correlated to vitamin E level. Nevertheless, in this study the greatest source of variation in MN induction was not attributed to absorbed dose, radioiodine uptake, basal thyroid volume, age, or sex, suggesting that other factors might influence MN induction and CF formation, such as polymorphic genes encoding enzymes involved in reactive oxygen species detoxification and DNA repair [18]. In a later study Dardano et al. [19] demonstrated a possible protective effect in genotoxic damage after ^131^I therapy with the administration of* Ginkgo biloba* extract (EGb 761). Intriguingly basal frequencies of MN were increased in both groups (placebo and EGb 761) when compared to the previous study, as well as the result at the 7th day but not at 21th day in the group receiving EGb 761. In both groups after 120 days MN frequency returned to basal values. In conclusion ^131^I therapy induces a significant, although transient, genetic damage characterized by an increase in MN frequencies. Authors suggest that the administration of EGb 761 can decrease this transient genetic damage [19]. A third study performed by Shanmuga-Sundaram et al. in 2011 [20] reported an analysis of peripheral blood lymphocytes cytotoxicity, in 32 hyperthyroid patients after the administration of radioactive iodine. This study corroborated previous findings, with a positive correlation between ^131^I dose and MN frequency, a significant increase in MN frequency at day 7th after therapy, and a decline after the 30th day after therapy. The authors concluded that since the cytotoxicity is transient and reversible, patients can be motivated to undertake ^131^I as an easy and safe treatment for hyperthyroidism [20]. Nevertheless in this study the authors did not provide information about baseline values of MN; it would have been interesting to know how much the autoimmune pathology itself can contribute to the generation of MN when compared to other inflammatory conditions.

Diabetes mellitus (DM) is characterized by an elevation in blood glucose that eventually is associated with excessive concentration in free radicals and oxidative damage to different biomolecules, and importantly the DNA causing strand breaks [37]. Zúñiga-González et al. in 2007 found an increased frequency of MN formation in patients with DM; this frequency was approximately twofold when compared to healthy subjects (*p* < 0.001) and was increased in type 2 DM (T2DM) when compared to type 1 (T1DM). Interestingly, the frequency of MN was significantly decreased after 30 days of folic acid supplementation (*p* < 0.001) [21]. T1DM is a complex autoimmune disorder caused by absolute deficiency of insulin due to destruction of pancreatic *β*-cells. Cinkilic et al. in 2009 evaluated the frequency of MN in T1DM patients and compared to healthy subjects, they found no differences in chromosomal aberrations or MN frequency. However they were able to demonstrate a significantly higher sister chromatids exchange frequency in T1DM when compared to healthy subjects, which may be due to generation of oxygen-derived free radicals occurring in this condition [22].

Multiple sclerosis (MS) is a chronic autoimmune disease, in which myelin-reactive lymphocytes can be found in the central nervous system. Four types of MS have been characterized: relapsing-remitting (RRMS), secondary progressive (SPMS), primary progressive (PPMS), and progressive relapsing (PRMS). The RRMS form is present in 85% of patients at disease onset, but later during the course most of them will develop the SPMS form [24]. We found two studies about MN in MS patients; they were performed to evaluate the resistance to radiation of lymphocytes from MS patients; resistance to radiation is defined as the capacity of lymphocytes to prevent the formation of MN after radiation. The first study was performed by Petcu et al. in 2006 [23]. They found that the spontaneous MN frequency was significantly higher in SPMS patients compared to healthy controls; such differences were attributed mainly to the increased level in oxidative stress and decreased antioxidant capacity in serum from MS patients. They also evaluated the radio sensitivity (*γ*-irradiation) by mean of MN formation in peripheral blood total lymphocytes, CD4+, and CD8+ T cells from SPMS patients compared to healthy controls; when analyzing total lymphocytes and CD8+ subset of cells, no differences were found, but a decreased radiosensitive response was reported in CD4+ T cells from MS patients. When patients and healthy subjects were analyzed before and after irradiation the induction of MN was evident, although frequencies tended to be higher in healthy subjects after radiation; this fact could be the consequence of a defective proliferation rate in patients with MS or can be the reflection of MS therapies that render CD4+ T cells resistant to MN formation after radiation when compared to cells derived from healthy subjects. Lately in 2013, Milenkova et al. [24] reported that immunomodulatory therapy with IFN-*β* or glatiramer acetate in RRMS patients reduces the number of MN in binucleated cells induced by* in vitro γ*-irradiation. This data suggests that the resistance to radiation by T lymphocytes in MS patients is mainly due to treatment with immunomodulatory agents. The characterization of particular sets of cells derived from MS patients is needed to further clarify their cytophatic effects, since lymphocytes in MS and actually in any AD have different characteristics that may influence cellular responses, such as higher number of cells with the Th1 profile with increased production of proinflammatory cytokines, impaired apoptotic deletion (increased production of proliferative cytokines and growth factors, such as IL-2 and upregulation of survivin), and modified T helper/T suppressor ratio [24]. Finally the mechanism of action of IFN-*β* is downregulation of vascular endothelial cells, decreasing the production of adhesion molecules and metalloproteases which influences migration of T cells, and inhibition of the generation of proinflammatory cytokines (TNF-*α*, IFN-*γ*, and IL-12). All together, these data suggest that therapy with IFN-beta could decrease the oxidative damage, making cells less sensitive to radiation in terms of MN formation.

Generalized vitiligo is an autoimmune disorder characterized by acquired white patches of skin and overlying hair, as a result of loss in melanocytes function. Several theories have been proposed to explain the pathogenesis of vitiligo, including self-destructive, biochemical, neural, autoimmune, and genetic hypotheses, but each of them is only applicable to a small proportion of cases [25]. High frequencies of skin-homing melanocyte-specific cytotoxic T lymphocytes in the peripheral blood of patients with vitiligo have been reported, suggesting melanocyte death in vitiligo. The autocytotoxic hypothesis suggests that melanocyte impairment could be related to increased oxidative stress. Additionally patients that suffer from vitiligo have decreased concentrations of holotranscobalamin, vitamin B12, and folates [38]. It has been described previously that these micronutrients are important for the maintenance of genome integrity [39]; therefore, we believe that oxidative stress along with folate deficiency could explain genomic instability in patients with vitiligo. This conclusion was demonstrated by Donmez-Altuntas et al. in 2008 [25], since they found significantly higher frequencies of MN in lymphocyte cultures of patients with vitiligo compared with control subjects, which points towards the use of MN as biomarkers during autoimmune conditions.

Psoriasis vulgaris is a common, chronic, proliferative, and recurrent inflammatory skin disease, with lesions that appear as a result of hyper proliferation, abnormal differentiation of keratinocytes, and epidermis infiltration with inflammatory cells including T cells and neutrophils. The etiology of psoriasis involves genetic, immune, and environmental factors [26, 40]. To evaluate cytotoxic and genotoxic damage in psoriasis patients after treatment, Silva et al. [26] performed a study in culture lymphocytes from psoriasis patients who received different treatment regimens: acitretin alone, acitretin + NBUVB (Narrow-band ultraviolet B irradiation), and acitretin + PUVA (psoralen + ultraviolet A irradiation). Results showed that at T0 all patients and controls presented similar MN frequencies, at T12 (12 weeks after the administration of treatments), both “acitretin + NBUVB” and “acitretin + PUVA” showed a trend towards an increase in MN frequency; however, only “acitretin + PUVA” reached a significant higher MN frequency. Authors state that the presence of psoriasis induced an increase in MN frequencies, and these frequencies were increased after 13 weeks of treatment with acitretin + PUVA [26].

### 3.2. Systemic Autoimmune Diseases

With respect to systemic autoimmune diseases, we found 2 published papers related to genotoxicity in rheumatoid arthritis (RA), 5 in systemic lupus erythematosus (SLE), 5 for systemic sclerosis (SSc), and 4 related to Behçet's disease.

Rheumatoid arthritis (RA) is a chronic systemic autoimmune condition that often presents with joint inflammation and destruction; furthermore, several studies have estimated the risk of malignancy in RA patients, with potential attributable risk factors such as therapies, mainly disease-modifying antirheumatic drugs (DMARDs), and the influence of RA itself. Ramos-Remus et al. in 2002 [12] evaluated the presence of MN in RA patients and compared them to healthy controls. They also performed a subanalysis within the RA population and search for an increased frequency of MN within subjects taking methotrexate (MTX) as part of their treatment. Authors found an increased baseline frequency of MN in RA patients when compared to healthy subjects, but the frequency was not significantly higher in RA patients taking MTX; and although it was previously demonstrated in an* in vitro* study that folic acid could inhibit the induction of MN by MTX, Ramos-Remus et al. found that the MN number did not decrease after folic acid supplementation, at least at the dose of 5 mg p.o. per day for 6 weeks. Authors concluded that the increase in MN frequency, observed in this study, is mainly due to RA itself rather than to MTX use. A subsequent study performed in 2011 by Karaman and coworkers [27] with the objective to determine if genetic lesions and DNA damage have an effect on the pathogenesis of RA tested the MN frequency in peripheral lymphocyte cultures; authors found that the number of MN/1000 BNCs (binucleated cells) was significantly higher in RA compared with controls.

Systemic lupus erythematosus (SLE) is a chronic autoimmune disorder that may affect diverse organs, characterized by the presence of antibodies against nuclear antigens (ANA) with different specificities (ssDNA, dsDNA, RNA, and snRNP among others); treatment for this condition includes diverse regimens of high dose steroids and immunosuppressive therapy. We found four studies related to MN frequency in SLE. The first study included in this review was performed by Severin in 1995 [28]. She analyzed the frequency of MN cells in peripheral blood lymphocytes and found the highest frequencies among SLE patients who had received cyclophosphamide for treatment, in comparison to patients with SLE without medical treatment and control subjects. This higher frequency was observed after 48 hours from the beginning of treatment. Later in 1999 Migliore et al. [4] performed a study in SLE and systemic sclerosis (SSc) patients, with the aim to assess the spontaneous frequency of cytogenetic damage in lymphocytes, using the MN assay; they found that the MN frequency did not differ between SLE and controls. A third study performed by Rodríguez-Vázquez et al. in 2000 [13] had the objective to evaluate MN frequencies in SLE patients taking cyclophosphamide (CYC) treatment and compare them to healthy controls; for this purpose they obtained buccal mucosa samples from 10 SLE patients and 43 healthy subjects; samples were obtained before treatment and fourteen days after the administration of CYC. They found that the frequency was similar in both groups at baseline, but in SLE 14 days after CYC administration the frequency in MN was significantly increased and maintained elevated before the second administration of CYC, with the highest frequency of MN observed 14 days after the second administration of CYC. The last study about MN frequency in SLE included in this revision was performed by Aceves-Avila et al. in 2004 [29]. They analyzed the effect of CYC and the influence of CYP2D6 polymorphisms on MN formation in patients with SLE; they found that the genotoxicity is increased in patients with SLE after CYC boluses without an association with the CYP2D6 allele expression, which contributes to the idea that irrespective from treatments patients with AD exhibit higher MN formation when compared to healthy subjects. The most recent study in this field was published in 2014 by Al-Rawi et al. [30]; after assessing nuclear morphology in 58 SLE patients and 58 healthy age- and sex-matched controls by the use of buccal micronucleus cytome assay, they proposed a score with a cutoff value of ≥4 that exhibited a high accuracy (93.1%), higher positive predictive value (98.1% at 50% pretest probability and 99.8% at 90% pretest probability), sensitivity of 87.9%, and specificity of 98.3% for the diagnosis of SLE. After these findings, they concluded that the buccal micronucleus cytome assay was a valid and easy way for clinicians to assess individuals at high risk for developing SLE, since a negative result in the total micronuclei count excluded a possible diagnosis of SLE with 98.9% confidence in a clinical setting in which the differential diagnosis of SLE was of low probability (10% pretest probability). Importantly this is the first report that analyzes MN frequency in an AD and proves the potential that this test has to be used as a biomarker of DNA damage in SLE.

Systemic sclerosis (SSc) is an autoimmune condition where the presence of antibodies against the centromere or directed to topoisomerase I (Scl70 antigen) can be found [4]. This systemic connective tissue disease is characterized by progressive fibrosis of the skin and internal organs; the presence of antinuclear antibodies (ANA) is frequently found in the serum with different specificities for nuclear targets [41]. Based on the extension of cutaneous sclerosis, SSc can be distinguished as a limited or diffuse; in most cases Raynaud's phenomenon (RP) is an early symptom of SSc that can precede the appearance of SSc by several years [31]. In 1999 Migliore et al. [4] performed an analysis of MN frequency in SSc patients and compared them to SLE and control subjects. Noteworthy, MN frequency was significantly higher in SSc patients when compared to both controls and SLE patients, especially in patients who were anti-centromere antibodies (ACA) positive and anti-Scl70 negative, indicating that the presence of autoantibodies against nuclear targets could account for chromosome anomalies which have been reported since 1960 in association with SSc. Porciello et al. in 2002 [42] described a first study in SSc patients; nevertheless this study was replicated in 2003 with a higher number of patients and with similar results [31]; for this reason we only included results from the latter study in the present review. They evaluated the prevalence of spontaneous chromosome damage, by means of MN assay, in cultured peripheral lymphocytes of patients with SSc (*n* = 43), idiopathic RP (*n* = 13), suspected secondary RP (*n* = 16), and healthy controls (*n* = 25). Patients were also classified in terms of autoantibody presence (ANA, ACA, or Scl70). They found that patients with SSc and subjects with secondary RP showed significantly higher MN frequencies than controls. Furthermore, ACA positive patients showed the highest MN frequencies, which suggest a possible role of ACA in determining cytogenetic anomalies [31]. Confirming the results of this previous study, Majone and coworkers described in 2007 [43] a significantly higher frequency of MN in SSc patients who were positive for ACA compared to SSc patients who were positive for antitopoisomerase or anti-RNA polymerase III as well as healthy controls. More importantly, they examined the nature of chromosome damage within analyzed lymphocytes for the presence of MN. Following the use of the in situ DIG-dUTP incorporation method using TdT enzyme, which indicates deep genetic instability [44], they were able to show that the frequency of unstable fragments of DNA (free 3′-OH ends) was significantly higher in SSc patients who were positive for ACA and antitopoisomerase, with the highest frequencies observed in ACA positive patients. Importantly, this genetic abnormality was not found in SSc patients with anti-RNA polymerase III antibody (*n* = 8), who had a low frequency of MN, similar to that in normal subjects [43]. The most recent study included in this review with insights into MN frequency in SSc patients was published by Martins et al. in 2010 [32]; the objective of their study was to evaluate chromosome damage, by means of MN frequency, in dermal fibroblasts from affected, nonaffected skin from SSc patients (*n* = 10) and from controls (plastic surgery remnants; *n* = 9); all of the cultures were analyzed before and after the administration of bleomycin. They found that the frequency of MN was higher in cells from SSc patients, irrespective of the area they were derived from, when compared to controls. In bleomycin treated cultures, the MN frequency was increased in all groups but patterns were kept, with the highest frequencies in cells derived from SSc patients. The authors conclude that increased chromosomal damage can affect dermal fibroblast from clinically affected and nonaffected skin in SSc patients, which may be related to oxidative stress derived from repetitive post-ischemic reperfusion episodes occurring in the tissues of SSc patients [32].

Behçet's disease (BD) is a chronic, multisystem, inflammatory disorder characterized mainly by recurrent oral and genital aphtha's ulcerations, uveitis, and erythema nodosum; although it can occur worldwide, it is most frequently seen in Turkey and Japan [35]. The first study included in this review was published in 2005 by Hamurcu et al. [33], with an evaluation of MN frequency in peripheral lymphocytes from 30 patients with BD and 20 healthy controls and on buccal mucosa cells in 10 patients and 9 healthy controls. They found significantly higher MN rates in lymphocytes derived from patients compared to controls and no difference in MN frequency of buccal mucosa cells, even when a subgroup of patients was exposed to the treatment colchicine. Later in 2009 Karaman et al. [34] performed a study to determine MN and sister chromatid exchange (SCE) frequencies in BD patients and to evaluate possible associations between oxidative stress and chromosome instability in BD. For this purpose lymphocyte cultures derived from 30 patients with BD and 20 healthy subjects were evaluated. Main results in this study include a significantly higher frequency of SCE in BD patients when compared to healthy subjects, and even a significantly higher frequency was observed in active patients (having at least three of the four major symptoms: oral aphthae, genital ulceration, cutaneous lesions, and uveitis), when compared to inactive patients. Different from SCE, the MN rate was significantly higher in BD patients when compared to healthy subjects, but there was no significant difference between active and inactive BD patients. Furthermore, the presence of oxidative stress was confirmed mainly in patients with active BD. These differences are important, since genetic abnormalities related to SCE are indicative of an early biological effect that may not be permanent and may not have further consequences, while MN presence represents early but irreversible biological effects. Authors conclude that the elevated rates of SCE and MN in BD patients can be explained by increased oxidative stress. Several studies have shown a significant association of human leukocyte antigen- (HLA-) B51 with BD, and for this reason Binici et al. in 2013 [35] aimed to investigate whether patients with the HLA-B51 allele had higher MN frequencies; after analyzing MN frequencies in lymphocytes from 40 patients with BD and 30 healthy controls, they found that the MN frequency was significantly increased in BD patients and this frequency was higher in HLA-B51 positive patients when compared to BD patients without this leukocyte antigen molecule.

## 4. Conclusion

It has been documented that AD with local or systemic affection are associated with genetic material loss; the presence of inflammatory cytokines, reactive oxygen species, and nitric oxide is involved in the pathology of AD as well as in the generation of MN, since the excess amount of these substances may show cytotoxic and genotoxic effects. This review provides strong information that sustains the observation that increased MN frequencies can be observed in association with the presence of any AD condition. On the other hand, treatments frequently used in AD can induce chromosomal damage as appeared evident in this review; such damage can be identified with an increased frequency of MN, since their presence provides an indirect and sensitive measure of chromosomal breakage or missegregation. Therefore the MN assay can be used to identify potential biomarkers that will allow the identification of early genotoxic damage in a simple and effective manner, with importance in early diagnosis, as well as an important tool for the differentiation of patients with different AD from healthy controls in suspected cases. Of consideration, after analyzing the information provided here we were able to observe that MN frequencies tend to be higher when lymphocytes are examined instead of buccal mucosa cells, irrespective of the disease evaluated. Furthermore, lymphocytes MN frequencies have higher variability that is independent of the presence of an AD; just by examining healthy subjects different frequencies were obtained (from 0.6 ± 0.3 to 27 ± 3.5). These discrepancies should be minimized in order to be able to extrapolate results in different populations and diseases. More important is the evidence that in all AD that were included in this review the presence of genomic instability is frequent when compared to healthy subjects, even though it is not known if it is a cause or a consequence of the disease. Another important observation is the fact that MN frequencies tend to be higher in systemic AD when compared to local affections; in fact RA and SSc had the highest MN frequencies even without the exposition to treatments. Finally, more studies that involve different populations of patients with other AD are needed to further clarify the mechanism of MN production in association with specific autoimmune conditions, since in some reports associations with specific autoantibodies could be detected. The influence of therapies on MN induction should be also clarified to give a correct interpretation of MN as early biomarkers of genomic damage with possible prognostic significance.

## Figures and Tables

**Table 1:** Evaluation of micronucleus (MN) frequencies in organ specific and systemic autoimmune diseases.

Disease	Cells analyzed	*N*	Study groups	MN		Year/reference
				Basal	Posttreatment	
Organ specific autoimmune diseases						

Graves' disease (GD)	Ly 2000 cells	11	GD + vit. E, after ^131^I	4.0 ± 3.2	10.3 ± 1.7 (7 d)^c^ 13.7 ± 2.3 (21 d) 3.9 ± 2.2 (180 d)	2004 [18]
	Ly 2000 cells	15	GD + placebo after ^131^I	5.2 ± 1.0	12.5 ± 1.6 (7 d) 24.7 ± 3.6 (21 d)^c^	2007 [19]
		10	GD + *Ginkgo biloba* after ^131^I	8.7 ± 1.8	14.3 ± 2.2 (7 d) 12.4 ± 1.9 (21 d)	
	Ly NA	25 25	GD 7 days after ^131^I GD 30 days after ^131^I	NA NA	2.4 ± 1.4% 1.9 ± 0.2%^a^	2011 [20]

Diabetes mellitus (DM)	BM 2000 cells	55 23 30 81	T1DM T2DM DM + folic acid Healthy subjects	2.4 ± 1.4^b^ 2.6 ± 1.6 2.5 ± 1.4 1.2 ± 1.2	0.7 ± 0.6^c^ (30 d)	2007 [21]
	Ly 2000 cells	35 15	T1DM Healthy subjects	1.8 ± 1.2 1.5 ± 0.6		2009 [22]

Multiple sclerosis (MS)	Ly 1000 cells	23 20	MS + radiation Healthy subjects	45.3 ± 3.3 27 ± 3.5	381 ± 16^a^ 347 ± 13	2006 [23]
	Ly 1000 cells	18 18 20	RRMS + radiation SPMS + radiation Healthy subjects + radiation	14.2 ± 6.1 12.7 ± 4.4 15.2 ± 6.5	86.0 ± 15.9^a^ 86.6 ± 22.8^a^ 96.7 ± 19.4^a^	2013 [24]

Vitiligo	Ly 1000 cells	21 21	Vitiligo without treatment Healthy subjects	0.9 ± 0.6%^a^ 0.6 ± 0.3%		2008 [25]

Psoriasis	Ly 1000 cells	5 6 5 5	P + acitretin (oral) alone P + acitretin + NBUVB Acitretin + PUVA Healthy subjects	NA NA NA NA	NS NS a NA	2013 [26]

Systemic autoimmune diseases						

Rheumatoid arthritis (RA)	BM 2000 cells	29 50 40 39	RA without MTX RA with MTX RA + MTX + folic acid Healthy subjects	3.5 ± 1.5 3.1 ± 3.1 3.1 ± 2.9 0.8 ± 0.8	3.1 ± 2.8	2002 [12]
	Ly 1000 cells	12 31 30	Active RA Inactive RA Healthy subjects	3.3 ± 1.3^a^ 3.2 ± 1.1^a^ 1.9 ± 1.1		2011 [27]

Systemic lupus erythematosus (SLE)	Ly 2000 cells	NA	SLE without treatment SLE with CYC Healthy subjects	6.2 ± 2.4 10.1 ± 4.7^c^ 5.5 ± 2.2		1995 [28]
	Ly 2000 cells	15 18	SLE without treatment Healthy subjects	10.6 ± 3.9% 9.1 ± 4.6%		1999 [4]
	BM 1000 cells	10 10 7 7 43	SLE before first CYC bolus SLE 14 days after 1st CYC bolus SLE before second CYC bolus SLE 14 after the 2nd CYC bolus Healthy subjects	NA	1.5 ± 1.6 2.9 ± 2.1 4.5 ± 2.3^a^ 7.0 ± 2.8^b^ 1.3 ± 1.3	2000 [13]
	BM 1000 cells	25 24 43	SLE without CYC SLE with CYC Healthy subjects	0.9 ± 1.1 1.3 ± 1.2 1.3 ± 1.4	3.4 ± 2.2 (14 d)^c^	2004 [29]
	BM 1000 cells	58 58	SLE patients Healthy controls	Median cell counts of MN were significantly higher among SLE patients		2014 [30]

Systemic sclerosis (SSc)	Ly 2000 cells	11 5 6 18	SSc SSc (ACA+/Scl70−) SSc (ACA−/Scl70+) Healthy subjects	27.4 ± 19.9^b^ 38.0 ± 24.9^b^ 18.7 ± 9.5^b^ 9.1 ± 4.6		1999 [4]
	Ly 1000 cells	43 13 16 25	SSc Idiopathic RP Secondary RP Healthy subjects	19.1 ± 2.1 23.5 ± 2.7 9.4 ± 2.2 25.9 ± 1.7		2003 [31]
	Fibroblasts 1000	10 10 9	SSc (nonaffected skin) + bleomycin SSc (affected skin) + bleomycin Healthy subjects + bleomycin	15.4 ± 13.6 14.0 ± 12.0 4.7 ± 3.3	38.5 ± 17.9^a^ 38.0 ± 26.1^a^ 20.5 ± 13.1	2010 [32]

Behçet's disease (BD)	BM 1000 cells	10 6 4 9	Patients with BD BD treated with colchicine BD without colchicines Healthy subjects	1.9 ± 0.9 1.7 ± 1.0 2.3 ± 0.5 0.8 ± 0.7		2005 [33]
	Ly 1000 cells	30 10 20 20	Patients with BD BD without colchicine BD with colchicine Healthy subjects	3.4 ± 1.1 3.6 ± 1.2^a^ 3.4 ± 1.1^a^ 2.2 ± 0.7		
	Ly 1000 cells	14 16 20	BD inactive BD active Healthy subjects	3.1 ± 1.3^b^ 3.2 ± 1.2^a^ 2.2 ± 0.7		2009 [34]
	Ly 1000 cells	18 22 30	BD negative HLA-B51 BD positive HLA-B51 Healthy subjects	2.6 ± 0.7^c^ 3.5 ± 0.7^c^ 1.7 ± 0.3		2013 [35]

*n*: sample size; d: days; statistical differences: ^a^ *p* < 0.05, ^b^ *p* < 0.01, and ^c^ *p* < 0.001; Ly: lymphocytes; BM: buccal mucosa; T1DM: type 1 diabetes mellitus; T2DM: type 2 diabetes mellitus; P: psoralen; PUVA: psoralen + UV A ultraviolet A irradiation; NBUVB: narrow-band ultraviolet B irradiation; MS: multiple sclerosis, RA: rheumatoid arthritis, MTX: methotrexate, SLE: systemic lupus erythematosus, CYC: cyclophosphamide, SSc: systemic sclerosis, ACA: anti-cytoplasmic antibodies, Scl-70: antibodies against topoisomerase I, RP: Raynaud's phenomenon, BD: Behçet's disease, NA: not available, NS: not significant.

## References

[1] Matzinger P. (2002). The danger model: a renewed sense of self. *Science*.

[2] Farwell A. P., Braverman L. E. (1996). Inflammatory thyroid disorders. *Otolaryngologic Clinics of North America*.

[3] Cabiedes J., Núñez-Álvarez C. A. (2010). Antinuclear antibodies. *Reumatologia Clinica*.

[4] Migliore L., Bevilacqua C., Scarpato R. (1999). Cytogenetic study and FISH analysis in lymphocytes of systemic lupus erythematosus (SLE) and systemic sclerosis (SS) patients. *Mutagenesis*.

[5] Türsen Ü. (2012). Pathophysiology of the Behçet's disease. *Pathology Research International*.

[6] Karaman A., Binici D. N., Melikoĝlu M. A. (2011). Comet assay and analysis of micronucleus formation in patients with rheumatoid arthritis. *Mutation Research—Genetic Toxicology and Environmental Mutagenesis*.

[7] Bonassi S., Coskun E., Ceppi M. (2011). The HUman MicroNucleus project on eXfoLiated buccal cells (HUMN XL): the role of life-style, host factors, occupational exposures, health status, and assay protocol. *Mutation Research*.

[8] Kashyap B., Reddy P. S. (2012). Micronuclei assay of exfoliated oral buccal cells: means to assess the nuclear abnormalities in different diseases. *Journal of Cancer Research and Therapeutics*.

[9] Schmid W. (1975). The micronucleus test. *Mutation Research*.

[10] Migliore L., Cocchi L., Scarpato R. (1996). Detection of the centromere in micronuclei by fluorescence in situ hybridization: its application to the human lymphocyte micronucleus assay after treatment with four suspected aneugens. *Mutagenesis*.

[11] Afshari A. J., McGregor P. W., Allen J. W., Fuscoe J. C. (1994). Centromere analysis of micronuclei induced by 2-aminoanthraquinone in cultured mouse splenocytes using both a gamma-satellite DNA probe and anti-kinetochore antibody. *Environmental and Molecular Mutagenesis*.

[12] Ramos-Remus C., Dorazco-Barragan G., Aceves-Avila F. J. (2002). Genotoxicity assessment using micronuclei assay in rheumatoid arthritis patients. *Clinical and Experimental Rheumatology*.

[13] Rodríguez-Vázquez M. S.-O., Ramos-Remus A., Zúñiga C., Torres-Bugarín O. (2000). Evaluación de la genotoxicidad de la ciclofosfamida mediante prueba de micronúcleos en pacientes con lupus eritematoso sistémico. *Revista Mexicana de Reumatología*.

[14] Heddle J. A., Cimino M. C., Hayashi M. (1991). Micronuclei as an index of cytogenetic damage: past, present, and future. *Environmental and Molecular Mutagenesis*.

[15] Torres-Bugarín O., Ventura-Aguilar A., Zamora-Perez A. (2003). Evaluation of cisplatin + 5-FU, carboplatin + 5-FU, and ifosfamide + epirubicine regimens using the micronuclei test and nuclear abnormalities in the buccal mucosa. *Mutation Research—Genetic Toxicology and Environmental Mutagenesis*.

[16] Holland N., Bolognesi C., Kirsch-Volders M. (2008). The micronucleus assay in human buccal cells as a tool for biomonitoring DNA damage: the HUMN project perspective on current status and knowledge gaps. *Mutation Research*.

[17] Ceppi M., Gallo F., Bonassi S. (2011). Study design and statistical analysis of data in human population studies with the micronucleus assay. *Mutagenesis*.

[18] Ballardin M., Barsacchi R., Bodei L. (2004). Oxidative and genotoxic damage after radio-iodine therapy of Graves' hyperthyroidism. *International Journal of Radiation Biology*.

[19] Dardano A., Ballardin M., Ferdeghini M. (2007). Anticlastogenic effect of *Ginkgo biloba* extract in Graves' disease patients receiving radioiodine therapy. *Journal of Clinical Endocrinology and Metabolism*.

[20] Shanmuga-Sundaram P., Padma S., Sudha S., Sasikala K. (2011). Transient cytotoxicity of ^131^I beta radiation in hyperthyroid patients treated with radioactive iodine. *Indian Journal of Medical Research*.

[21] Zúñiga-González G. M., Batista-González C. M., Gómez-Meda B. C. (2007). Micronuclei in diabetes: folate supplementation diminishes micronuclei in diabetic patients but not in an animal model. *Mutation Research*.

[22] Cinkilic N., Kiyici S., Celikler S. (2009). Evaluation of chromosome aberrations, sister chromatid exchange and micronuclei in patients with type-1 diabetes mellitus. *Mutation Research*.

[23] Petcu I., Savu D., Thierens H., Nagels G., Vral A. (2006). In vitro radiosensitivity of peripheral blood lymphocytes in multiple sclerosis patients. *International Journal of Radiation Biology*.

[24] Milenkova M., Milanov I., Kmetska K. (2013). Chromosomal radiosensitivity in patients with multiple sclerosis. *Mutation Research*.

[25] Donmez-Altuntas H., Sut Z., Ferahbas A., Hamurcu Z., Demirtas H. (2008). Increased micronucleus frequency in phytohaemagglutinin-stimulated blood cells of patients with vitiligo. *Journal of the European Academy of Dermatology and Venereology*.

[26] Silva F. S. G., Oliveira H., Moreiras A. (2013). Cytotoxic and genotoxic effects of acitretin, alone or in combination with psoralen-ultraviolet A or narrow-band ultraviolet B-therapy in psoriatic patients. *Mutation Research*.

[27] Karaman A., Binici D. N., Melikoĝlu M. A. (2011). Comet assay and analysis of micronucleus formation in patients with rheumatoid arthritis. *Mutation Research*.

[28] Severin E. (1995). Micronucleus frequency in peripheral blood lymphocytes from patients with systemic lupus erythematosus. *Romanian Journal of Morphology and Embryology*.

[29] Aceves-Avila F. J., Esquivel Nava G. A., Gallegos Arreola M. P., Gómez Meda B. C., Zúñiga González G. M., Ramos-Remus C. (2004). Cyclophosphamide boluses induce micronuclei expression in buccal mucosa cells of patients with systemic lupus erythematosus independent of cytochrome P450 2D6 status. *Journal of Rheumatology*.

[30] Al-Rawi Z. S., Gorial F. I., Tawfiq R. F. (2014). Brief report: a novel application of buccal micronucleus cytome assay in systemic lupus erythematosus: a case-control study. *Arthritis & Rheumatology*.

[31] Porciello G., Scarpato R., Ferri C. (2003). Spontaneous chromosome damage (micronuclei) in systemic sclerosis and Raynaud's phenomenon. *Journal of Rheumatology*.

[32] Martins E. P., Fuzzi H. T., Kayser C. (2010). Increased chromosome damage in systemic sclerosis skin fibroblasts. *Scandinavian Journal of Rheumatology*.

[33] Hamurcu Z., Dönmez-Altuntas H., Borlu M., Demirtas H., Asçioslu Ö. (2005). Micronucleus frequency in the oral mucosa and lymphocytes of patients with Behçet's disease. *Clinical and Experimental Dermatology*.

[34] Karaman A., Kadi M., Kara F. (2009). Sister chromatid exchange and micronucleus studies in patients with Behçet's disease. *Journal of Cutaneous Pathology*.

[35] Binici D. N., Karaman A., Kadi M. (2013). Micronucleus analysis in behçet's disease with and without HLA-B51. *Turkish Journal of Physical Medicine and Rehabilitation*.

[36] Effraimidis G., Wiersinga W. M. (2014). Mechanisms in endocrinology: autoimmune thyroid disease: old and new players. *European Journal of Endocrinology*.

[37] Dandona P., Thusu K., Cook S. (1996). Oxidative damage to DNA in diabetes mellitus. *The Lancet*.

[38] Montes L. F., Diaz M. L., Lajous J., Garcia N. J. (1992). Folic acid and vitamin B12 in vitiligo: a nutritional approach. *Cutis*.

[39] Fenech M. (2003). Genomic stability: a new paradigm for recommended dietary allowances (RDAs). *Forum of Nutrition*.

[40] Rucević I., Barisić-Drusko V., Glavas-Obrovac L., Stefanić M. (2009). Vitamin D endocrine system and psoriasis vulgaris—review of the literature. *Acta Dermatovenerologica Croatica*.

[41] van den Hoogen F., Khanna D., Fransen J. (2013). 2013 classification criteria for systemic sclerosis: an American College of Rheumatology/European League against Rheumatism collaborative initiative. *Arthritis and Rheumatism*.

[42] Porciello G., Scarpato R., Storino F. (2002). The high frequency of spontaneous micronuclei observed in lymphocytes of systemic sclerosis patients: preliminary results. *Reumatismo*.

[43] Majone F., Cozzi F., Tonello M. (2007). Unstabilized DNA breaks in lymphocytes of patients with different subsets of systemic sclerosis. *Annals of the New York Academy of Sciences*.

[44] Majone F., Luisetto R., Zamboni D., Iwanaga Y., Jeang K.-T. (2005). Ku protein as a potential human T-cell leukemia virus type 1 (HTLV-1) Tax target in clastogenic chromosomal instability of mammalian cells. *Retrovirology*.

